# The Role of Defense Mechanisms, Personality and Demographical Factors on Complicated Grief following Death of a loved one by Cancer

**Published:** 2015-04

**Authors:** Isaac Rahimian Boogar, Siavash Talepasand

**Affiliations:** 1 Department of Clinical Psychology, Faculty of Psychology & Educational Sciences, Semnan University, Semnan, Iran

**Keywords:** *Cancer*, *Character*, *Demographic*, *Grief*, *Personality*.

## Abstract

**Objective:** Identification of the risk factors and psychological correlates of prolonged grief disorder is vital for health promotions in relatives of persons who died of cancer. The aim of this research was to investigate the role of defense mechanisms, character dimension of personality and demographic factors on complicated grief following a loss of a family member to cancer.

**Method:** A number of 226 persons who had lost a family member to cancer in a cancer institute at Tehran University of Medical Science were selected through compliance sampling and completed the Inventory of complicated Grief-Revised (ICG-R), the Defense Styles Questionnaire (DSQ), the Character dimension of Temperament and Character Inventory (TCI), and the Demographical questionnaire. Data were analyzed by stepwise multiple regression analysis, using the PASW version 18.

**Results:** Findings revealed that neurotic defense style had a significant positive predictive role in the complicated grief; and cooperativeness, age of the deceased person, self-transcendence and mature defense style had a significant negative predictive role in complicated grief (p<0.001). R2 was 0.73 for the final model (p<.001).

**Conclusion:** The results revealed that two character dimensions (low cooperativeness and self-transcendence), high neurotic defense style and young age of the deceased person were involved in the psychopathological course of the complicated and prolonged grief. It was concluded that personality characteristics of the grieving persons and demographics of the deceased person should be addressed in designing tailored interventions for complicated grief.

Bereavement is a worldwide event, and the majority of persons suitably adjust with it. However, various researches have revealed that individuals experiencing prolonged bereavement have elevated disturbances, and they are at heightened risk for death (1,2). There is a demand to recognized susceptible bereaved persons so that treatments could reduce their risk of unfavorable consequences (2,3(.

Argument regarding the role of personality traits in comprehending and predicting psychopathological states is long-standing; and among the personal risk factors for these psychopathological phenomena, personality assumedly continues as a fundamental agent (4). Character and coping strategies including defense styles are the most prominent personal risk factors for complicated and prolonged grief (5, 6).

According to DSM-5 and ICD-11, complicated grief disorder (CGD) or prolonged grief disorder is the appearance of particular emotional, cognitive and behavioral symptoms in the past 6 months following the death of a loved one, including confusion concerning one's responsibility and roles, disability in confidence to members of the immediate family, difficulty in accepting the loss, extreme unpleasantness regarding the loss, disinterest to the others, mental or physical discomfort about life, absence of sentiment since the death, life's meaningless experiences and feeling stunned by the loss (3,7). Despite the importance of the study in this field, very little investigations have examined agents of complicated and prolonged grief pre- and post-loss. Moreover, these little investigations did not concentrate on the factors that anticipate complicated grief (8).

Multicultural and multiple factors in integrated manner may result in psychosocial stress, grief and mourning among family caregivers of younger and older patients with cancer (9-11). Some factors including personality traits and social background had proven to affect post-bereavement (12, 13).

Only few studies were conducted on the influences of defense mechanisms on the intensity of grief. On the other hand, more studies were performed about coping strategies instead of defense mechanisms to handle loss; this is because defense mechanisms are enduring tendencies and are mainly unconscious and so often resistant to change (14). However, it is important to study the effects of defense styles on complicated grief; interventional and therapeutic manners could be applied to change these maladaptive traits of personality (4).

Character structure is composed of three dimensions encompassing self-directedness (SD), which pertains to responsibility, self-acceptance and inclination toward goal setting; cooperativeness (C), which ascribes to the capacity to recognize and admire other people; self-transcendence (ST), which shows an inclination toward value system, spirituality and being as important part of natural world (15). The concept of character became extremely influential and greatly considered by numerous prominent studies of psychopathology, but few studies have been conducted on the Cloninger's model of personality for severity of grief (16, 17). Bridging this gap is the one purpose of this research in Iran. 

Beside defense mechanisms and character, socio-demographical factors also have an important role in the incidence of prolonged and complicated grief after death by cancer. For example, Ball concluded that style of death and age predicted the intensity of grief in widows whose husbands died of illness or accident six to nine months proceeding to the research (18). In previous studies, age was a significant predictor of grief intensity compared to style of death (18,19). Without consideration for the style of death, the younger widow experienced extreme grief compared to middle aged or older widows. In one related study, demographic and clinical factors predicted the preparatory grief in advanced cancer patients (19, 20). 

On the other hand, designing appropriate interventions for bereaved caregivers of cancer patients is a one necessary task (21, 22). The aim of this research was to clarify the relationships between defense styles, character dimension of Cloninger's psychobiological model of personality structure and complicated grief.

## Material and Methods

This cross-sectional study was carried out among the family members of cancer patients whose patient has already died in the cancer institute at the Tehran University of Medical Science between March 2012 to July 2012. Over the 5-month study period, 226 persons (108 male and 118 female) following the death of one family member by cancer at the average age of 2.89 (SD =1.83) after death were selected by compliance sampling. Death by cancer at least 6 months prior to the study among family members (parents or siblings), needlessness for present medical care, interest for study participation and full completion of self-report instruments were the inclusion criteria. In addition, all persons with severe psychopathological disorders who were diagnosed by a psychiatrist, surgical inpatients or outpatients, medical patients who were clearly moribund or in a coma after events such as cardiac arrest (n= 22) were excluded from the final analysis. Given the no interventional nature of this cross-sectional study, and in agreement with ethics committee demands at the inset of the research, informed consent was obtained from the participants and ethical considerations were addressed in the research. 

*Instruments*

Participants completed the Inventory of Complicated Grief-Revised (ICG-R). The Defense Styles Questionnaire (DSQ), The Character dimension of Temperament and Character Inventory (TCI) and The Demographic questionnaire.

Inventory of Complicated Grief-Revised (ICG-R) is an adjusted and brief version of the primary Inventory of Complicated Grief (ICG), which is composed of 19 items (23). The ICG-R was created to evaluate maladaptive manifestations of loss and includes all symptoms suggested for the Prolonged Grief Disorder diagnosis (24). Indeed, the ICG-R is established upon 15 questions and has a practical criterion and a time criterion of 6 months which is scored on a Likert scale (five-point). Because of the duration criterion of 6 months, the ICG-R was executed at 6 months as the initial evaluating point. The ICG-R reliability with inter-item correlation had verified by Danish study (0.52) and Cronbach’s >0.94 (25). Also, ICG-R contained the gold standard with appropriate cut off point for differentiating the normal grief from prolonged grief as at first proposed by the constructors of the scale (23). Utilizing this procedure, the cut off point for ICG-R in the Danish sample was a score of 43 and above. The ICG-R is a properly validated scale (α = 0.92– 0.94; test–retest reliability = 0.80 (23). Threshold level of the complicated grief manifestations was specified as a score ≥25 (26). Also, the validity and reliability (Cronbach’s α = 0.88) of the ICG-R were confirmed in Iranian nonclinical sample (27). This scale with proper psychometric features is useful for measuring the severity of prolonged and complicated grief (28).

Defense Styles Questionnaire (DSQ) contained 40 items about a broad diversity of defense mechanisms, which are organized into three extensive defense styles: mature (8 items), immature (8 items), and neurotic (24 items) (29). This scale is a self-report instrument in which participants respond by a nine-point Likert scale extending from one (strongly disagree) to nine (strongly agree). Thus, elated scores point out higher utilization of the target defense style. Andrews et al. stated that alpha coefficient amounts of the Defense Styles Questionnaire scales extend from .58 (neurotic style) to .80 (immature style) (29). Cronbach's alpha estimates in a study by Gana and K'Delant were .65, .66 and .83 for mature, neurotic and immature styles, respectively (4). Factor analysis of Defense Styles Questionnaire (DSQ-40) in Iranian nonclinical sample revealed an excellent validity of this scale (30). Moreover, reliability coefficients of Cronbach's alpha for mature style, neurotic style and immature style was α = 0.75, α = 0.73 and α = 0.74, respectively which confirms suitable reliability of this scale (30).

The Temperament and Character Inventory (TCI) is a force-choice and true-false self-report instrument to evaluate individual distinctions in the fundamental dimensions of biosocial model of personality according to the Cloninger theory (31, 32). The TCI contains 226 items to measure seven dimensions reflecting about two major components of temperament and character of personality formulated to assess seven dimensions. Dimensions of temperament were harm avoidance (HA), novelty seeking (NS), reward dependence (RD) and persistence (P). Dimensions of character were self-directedness (SD), cooperativeness (C) and self-transcendence (ST). In this study, dimensions of character were used. In both normal and abnormal personality patterns, the one version of the TCI was used. Coefficients of internal consistency for the TCI scales by using Kuder–Richardson formula (K–R20) are higher than 0.70 (32). TCI was stable with Intraclass coefficients from 0.66 to 0.82 (P-0.001) and its reliability coefficients with Cronbach were above.75 (33). The psychometric characteristics of the TCI were affirmed by its reliability values and validity properties (33). The Temperament and Character Inventory (TCI) has appropriate psychometric features in Iranian population (34).

Demographic questionnaire was also used to gather data on the following factors: Age, gender, socio-economic status, marital status, literacy, relationship to the deceased person, location of the usual residence, history of trauma and age of the deceased person.

*Statistical Analysis*

Multiple regression analysis with stepwise procedure was used to reveal which of the factors included in this model significantly predicted complicated grief. This data analysis was performed using the PASW version 18. Multiple regression analysis is the best statistical method to investigate the predictive relationships among the predictors and continuous criterion variables (35). P value downwards from 0.05% was marked as significant. 

## Results

Participants' age ranged from 19 to 84 years with the mean age of 43.40 (SD = 11.40). Among all participants, 108 (47.8%) were male and 118 (52.2) were female. Also, 130 (57.5%) participants had a poor socio-economic level and 96 (42.5%) had a good socio-economic status. In addition, 122 (54.0%) participants were parents of a deceased person and 104 (46.0%) participants were siblings of a deceased person. Preliminary data analysis was carried out to ensure that no violation of the statistical assumptions of linearity, collinearity and multicollinearity, normality and homoscedasticity has occurred. Initially, bivariate correlations with Pearson calculation between whole of the variables used in the study were presented in Table 1. In this association, the correlation coefficients between the age of the deceased person, mature defense style, immature defense style, neurotic defense style, cooperativeness, self-directedness and self-transcendence with complicated grief was (r = -.57,P = .001), (r = -.59,P = .001), (r = .54,P = .001), (r = .71, P = .001), (r = -.66, P = .001), (r = -.60,P = .001), (r = -.54, P = .001), respectively.

Also, biserial correlation between the categorical predictors of gender and severity of complicated grief with r = .21 was statistically significant (P<.01). Significant predictors were entered to the regression model in the five steps. Neurotic defense style was entered in Step 1, explaining 50% of the variance in complicated grief (P<0.001). In step 2, neurotic defense style and cooperativeness were entered, explaining 64% of the variance in complicated grief (P<0.001). In step 3, neurotic defense style, cooperativeness and age of the deceased person were entered, explaining 69% of the variance in complicated grief (P<0.001). Neurotic defense style, cooperativeness, age of the deceased person and self- transcendence were entered in step 4, and together they explained 72% of the variance in complicated grief as a whole (P<0.001). After entry of all significant predictors (Neurotic defense style, cooperativeness, age of deceased person, self- transcendence and mature defense style) in step 5, the total variance in complicated grief was found by this model as a whole and it was 73%, P<.001, R squared change ( R2) = .01, F change 5,220 = 8.138, P<.001. 

Among all predictors (eight variables) entered to the model, the five predictive variables of the neurotic defense style, cooperativeness, age of the deceased person, self- transcendence and mature defense style significantly predicted the complicated grief. In this regression model using the stepwise method, R2 was .50, .64, .69, .72, and .73 for Steps 1, 2, 3, 4 and 5, respectively (p<.001). 

In this model, three variables of gender, immature defense style and self-directedness were excluded variables and did not have a significant role in predicting complicated grief (P>.05). A final regression formula for predicting complicated grief can be mentioned as follows: 

Complicated grief = 15.75(t) +.35(n)-.28(c)-.21(a)-.18(s)-.12 (m)

So that, t denotes statistical t-value, n denotes neurotic defense style, c denotes cooperativeness, a denotes age of deceased person, s denotes self-transcendence and m denotes mature defense style).

**Table 1:** Mean (Standard Deviation) and Correlation among the Continuous variables (N =226)

**Measure**	**1**	**2**	**3**	**4**	**5**	**6**	**7**	**8**
1. Age of deceased person								
2. Mature Defense Style	037*							
3. Immature Defense Style	-.32*	-.71**						
4. Neurotic Defense Style	-.42*	-.54**	.60**					
5 Cooperativeness	.38*	.49*	-.45*	-.46*				
6. Self- directedness	.34*	.44*	-.37*	-.40*	.84**			
7. Self- transcendence	029*	.33*	-.32*	-.38*	.41*	.36*		
8. Severity of Grief	-.57**	-.59**	.54**	.71**	-.66**	-.60**	-.54**	
Mean (Standard Deviation)	42.01 (12.91)	28.40 (13.34)	34.11 (15.19)	98.61 (16.32)	42.26 (21.00)	42.11 (19.42)	36.67 (12.89)	33.44 (15.17)

* Note. P<.01,

** P<0.001

**Table 2:** Regression Coefficients for Prediction of Complicated Grief in terms of defense mechanisms and personality (N =226)

		B	***SE*** B	β
Step 1	Constant	12.13	1.57	
	Neurotic Defense Style	0.197	.013	.71***
Step 2	Constant	31.11	3.33	
	Neurotic Defense Style	0.142	.012	.51**
	Cooperativeness	-0.309	.032	-.42**
Step 3	Constant	43.81	3.08	
	Neurotic Defense Style	0.121	.012	.43**
	Cooperativeness	-0.266	.031	-.368**
	Age of deceased person	-0.292	.049	-.248*
Step 4	Constant	50.73	3.27	
	Neurotic Defense Style	0.110	.012	.39**
	Cooperativeness	-0.228	.031	-.31*
	Age of deceased person	-0.267	.047	-.22*
	Self- transcendence	-0.227	.047	-.19*
Step 5	Constant	54.12	3.43	
	Neurotic Defense Style	0.098	.012	.35**
	Cooperativeness	-0.204	.031	-.28*
	Age of deceased person	-0.251	.047	-.21*
	Self-transcendence	-.218	.046	-.18*
	Mature Defense Style	-0.143	.050	-.12*

Note. R^2^=.50 for Step 1(P<0.001) and ∆R^2^ =.14, .04, .02, and .01 for Step 2, 3, 4 and 5 respectively (p<.05).

* p<.05,

** p<.01,

*** p<.001.

## Discussion

This study was intended to carefully look over the role of defense mechanisms, character of personality and socio-demographical factors on complicated grief among family members following a death due to cancer in a family. The influence of personality traits in comprehending grief complications and psychological intervention of complicated grief should be explored. Here, we have summarized the main results of this study. Firstly, according to the outputs of multiple regression analysis in the final model, the neurotic defense style had a significant positive influence on complicated grief and cooperativeness, age of deceased person, self- transcendence; and mature defense style had a significant negative effect on complicated grief. 

Neurotic defense mechanisms are maladaptive strategies for dealing with adverse realities. Watson, 

using the Brief Symptom Inventory (BSI), concluded that immature and neurotic defense styles were main 

predictors of the studied symptomatology (36). In line with prior studies, we can claim that maladaptive defense style such as neurotic defense mechanisms include susceptibility results in the complicated grief (4, 37). On the other hand, full formation of mature defense style is associated with lower tendency to experience complicated grief.

In this multiple regression analysis, age of the deceased person and the two character dimensions (cooperativeness and self-transcendence) were the other predictors of complicated grief. Therefore, as anticipated, death due to cancer in a younger age is a risk factor for prolonged and pathological grief. This result is consistent with previous literature in this field (38, 39).

The age of the deceased person negatively predicted complicated grief; this indicates that the younger age of the deceased person is associated with the severity of the grief. Obviously, it is more troublesome to admire the dying of a young person or one’s child. Also, death in the young is sometimes interpreted as a massive unfairness of fortune. Anyway, age is a relative and personal factor in grief-related experiences. 

In the personality dimensions, cooperativeness had a significant negative effect on complicated grief. Cooperativeness refers to empathy, compassion, social acceptance, kindliness and usefulness. Thus, existence of a high level of cooperativeness is associated with adoptive personality traits, and existence of a lower level of cooperativeness is an indicator of immaturity of character (40). Accordingly, grief psychopathology occurs when emotions are not regulated in a successful manner by a mature and full-grown character (4).

Moreover, self-transcendence was a negative predictor of complicated grief. This result requires some interpretation. Self-transcendence pertains to such traits as judiciousness, faithful and spirituality and these attributes seem to defend against persistence of grief, because these traits are beneficial when experiencing negative life events (41).

According to our results, gender, immature defense style and self-directedness do not significantly predict prolonged grief. In opposing results, a study by Gana and K'Delant revealed that gender, immature defense style and self-directedness were predominant predictors of prolonged grief (4). In accordance with bulk of researches, gender, immature defense style and self-directedness had proven to be determining factors in grief psychopathology (36, 42). Chiu and colleagues argued that gender and character dimensions of personality along with other factors are determinants of prolonged and complicated grief in persons who provide care and nursing to patients suffering from terminal cancer (43). It is argued that these factors were not significant predictors for prolonged and complicated grief in this study unlike the other studies, because of cultural differences, small sample size, different and self-report instruments, distinctive study design and diversity in methodology of the other studies. To better comprehend our results, the present research is the initial inquiry that attempted to inspect the associations between character dimension of personality and complicated grief in an Iranian sample. Therefore, subsequent studies in this field will reveal the greater facts in this regard.

## Limitation

It is obvious that this research had some drawbacks. Firstly, our questionnaire was extended causing a possible origin of bias due to fatigue, and it was settled by self-report items whose answers may be polluted by factors such as social desirability. Secondly, the sample size was small. Thirdly, some psychopathological disorders along with grief that were treated with psychotherapeutic interventions and drug treatments were not controlled. Therefore, a self-report scale in the assessment of complicated grief requires to be complemented by a clinical measurement. Eventually, investigating the associations between character, defense mechanisms and complicated grief can profit deeper understanding in the direction of what personality characteristics underlie grief severity and extended grief.

## Conclusion

According to the results, character dimensions including low cooperativeness and self-transcendence, neurotic defense style and lower age of the deceased person had shared in appearance of the complicated/prolonged grief. Eventually, we can infer that particular personality related elements with dysfunctional defense mechanisms may make individuals more susceptible to experience a prolonged or complicated grief disorder in loss of someone to cancer. Thus, the character dimensions of personality for bereaved persons and demographic characteristics of deceased persons should be addressed in future studies.
